# Detection of Virulence Determinants of Uropathogenic Escherichia coli

**DOI:** 10.7759/cureus.79116

**Published:** 2025-02-16

**Authors:** Nivetha R M, Shanthi Mariappan, Uma Sekar, Aishwarya K.V.L

**Affiliations:** 1 Microbiology, Sri Ramachandra Institute of Higher Education and Research, Chennai, IND

**Keywords:** antimicrobial resistance, cytotoxic necrotizing factor 1, e. coli, p fimbriae, upec, urinary tract infection, virulence factors, α-hemolysin

## Abstract

Background

*Escherichia coli* is the major causative agent of urinary tract infections (UTIs). Emergence and spread of multidrug-resistant strains of *E. coli* have raised considerable interest in understanding their diversity and epidemiology of infections in humans. Knowledge of virulence factors of *E. coli* responsible for pathogenesis of UTIs and their antibiotic susceptibility pattern will help in better understanding of the treatment of UTI. This study was undertaken to characterize virulence determinants of uropathogenic *E. coli *(UPEC) by genotypic methods and to determine their susceptibility to various classes of antibiotics.

Materials and methods

A total of 150 clinically significant, non-repetitive, consecutive *E. coli* isolated from urine were included in this study. Polymerase chain reaction (PCR) was done to detect the presence of virulence genes namely *fim H, iut A, hly A, pap C, cnf 1, *and s*at. *Antibiotic susceptibility testing was done by the disc diffusion technique according to Clinical and Laboratory Standards Institute (CLSI). The antibiotics tested were amikacin, gentamicin, fosfomycin, ampicillin, cefotaxime, ciprofloxacin, cotrimoxazole, nitrofurantoin, piperacillin-tazobactam and imipenem.

Results

Of the 150 *Escherichia coli*, 112 carried one or more of the virulence genes included in the study protocol. The genes *fim H (77%), iut A (57%), *and s*at (45%)* were the most common. The other genes include c*nf 1(26*%*)* and hl*y A (2%). Pap C* gene was not detected in any of the isolates*. *Among the 150 study isolates, the antimicrobial resistant pattern was 12.6% (19/150) for amikacin, 18.6% for gentamicin, 2% for fosfomycin, 88% for ampicillin, 67% for cefotaxime, 85% for ciprofloxacin, 62% for cotrimoxazole, 7.3% for nitrofurantoin and piperacillin-tazobactam, and 12% for imipenem. The frequency of sat gene was significantly higher in isolates resistant to gentamicin, ampicillin, cefotaxime, and cotrimoxazole. The occurrence of *iut A* was higher among isolates resistant to ampicillin, ciprofloxacin, cotrimoxazole, and piperacillin-tazobactam.

Conclusion

*fim* H is the most common virulence determinant among UPEC. *Pap* gene was not anchored in the study isolates. A combination of two or more virulence in single isolates was frequently encountered. Among the 150 study isolates, 68% were extended spectrum beta-lactamase producers and 12 % were carbapenem-resistant *Escherichia coli*. There was no significant association between the virulence gene and the antimicrobial resistance profile.

## Introduction

Urinary tract infections (UTIs) are one of the common bacterial illnesses that affect humans. According to the location of the infection, UTIs are categorized generally as pyelonephritis (the kidney) and cystitis (the bladder). The adhesion to host cells, colonization of tissues, and, in some cases, cellular invasion are necessary for the successful establishment of infection by bacterial pathogens. These steps are then followed by intracellular multiplication, dissemination to other tissues, or persistence. Uropathogenic *Escherichia coli* (UPEC) is the most common cause of UTI. A wide range of virulence factors are linked to UPEC for causing symptomatic UTIs. The bacterial cell surface and secreted virulence factor are the two broad categories into which the virulence factors of* E. coli* can be classified. These virulence factors include siderophores (aerobactin system), fimbrial adhesins (P, type 1, S, and F1C fimbriae), toxins (hemolysin and cytotoxic necrotizing factor), and capsular polysaccharide (group II capsules) [1]. Fimbriae, particularly type 1 fimbriae and P fimbriae are among the most frequent virulence factors found on bacterial cell surfaces. These fimbriae support adhesion to the host cell surface, cytokine induction, biofilm formation, and tissue invasion [2]. Adhesins aid in the adhesion of the organism to the surface of the epithelial cell, which allows it to avoid the flushing action that occurs during micturition. In addition to directly activating host and bacterial cell signaling pathways, the adhesins can also facilitate the delivery of other bacterial products to host tissues and encourage bacterial invasion. Type 1 pili or the f*im H *adhesin mediates bacterial attachment to several glycoproteins and non-glycosylated peptide epitopes in the bladder epithelium, which results in bacterial internalization and the formation of intracellular bacterial communities. The pap operon (pilus associated with pyelonephritis) codes for the various structural subunits of the P fimbriae [3,4].

Hemolysin, cytotoxic necrotizing factor type 1 (*cnf*1), and the secreted autotransporter toxin (s*at*) are the three types of toxins produced by UPEC. Hemolysin A (hl*y A*), also referred to as the "pore-forming toxin," enters the host cell membrane and causes cell lysis, which makes it easier for iron and other nutrients that are crucial for bacterial growth to be released. Cytotoxicity and invasiveness are the mechanisms by which hemolysin and cytotoxic necrotizing factor act. The erythrocytes are lysed, releasing nutrients and other vitamins that are then accessible to the bacteria. In addition, it releases inflammatory mediators and enzymes that damage the renal proximal tubular epithelium by being cytotoxic to erythrocytes, leukocytes, and renal proximal tubular epithelial cells [4]. *E. coli* produce siderophores like i*ut* A, which absorb iron from the host and aid in colonization and survival [3]. The s*at* toxin is a serine protease that belongs to the serine protease autotransporters of Enterobacteriaceae (SPATE), which is primarily found in strains of UPEC and is known for its cytopathic effects on the kidney and bladder. This toxin causes vacuolization in the cytoplasm of the uroepithelial cells [4]. Global data indicate that the pathogens that cause UTIs are becoming more resistant to traditional antimicrobial agents [1]. Even to the more recent and powerful antimicrobial agents, resistance has developed. To assess the severity of the issue and direct the empirical selection of antimicrobial agents to treat infected patients, antimicrobial resistance surveillance is required [1]. This study was undertaken to detect the genes encoding for virulence factors in UPEC and to determine their antimicrobial susceptibility profile.

## Materials and methods

Ethical approval

The present study was carried out at a 1600-bedded tertiary care, teaching hospital (Sri Ramachandra Institute of Higher Education and Research, Chennai, India). The methodology was approved by the Institutional Ethics Committee (for Medical PG Students) SRIHER (DU) (CSP-MED/20/OCT/62/112).

Bacterial isolates

The study included 150 clinically significant, non-consecutive, non-duplicate urinary isolates of* E. coli*, collected over a period of one year from November 2020 to October 2021. The isolates were identified by standard biochemical tests or by the automated VITEK-2 system (Vitek-2 GN-card; BioMerieux, Marcy-l'Étoile, France). 

Antibiotic susceptibility testing

Susceptibility testing for various antibiotics was done by the Kirby-Bauer disc diffusion method and interpreted in accordance with the Clinical and Laboratory Standards Institute guidelines (CLSI 2021). The antibiotics tested were amikacin (30mcg), gentamicin (10mcg), fosfomycin (200mcg), ampicillin (10 mcg), cefotaxime (30mcg), ciprofloxacin (5 mcg), cotrimoxazole (1.25/23.75), nitrofurantoin (300 mcg), piperacillin-tazobactam (100/10), and imipenem (10mcg). The antibiotic discs were procured from Himedia Laboratories (Mumbai, Maharashtra, India).

Template DNA preparation

A single bacterial colony was inoculated into Luria-Bertani broth (Himedia Laboratories, Mumbai, Maharashtra, India) and incubated at 37°C overnight, and centrifuged at 10,000 rpm for 10 minutes. The supernatant was discarded, and the pellet was resuspended in 250 μL of Millipore water, boiled at 100°C for 10 minutes in a water bath, and centrifuged at 10,000 rpm for 10 minutes. The supernatant is used as the template DNA.

Detection of virulence genes

A total of six virulence genes of *E. coli* were detected by polymerase chain reaction (PCR). The primers used for different virulence genes, their annealing temperature, and the amplicon size are listed in Table 1.

**Table 1:** The primers used for different virulence genes, their annealing temperature, and the amplicon size

Virulence genes	Primers (5’- 3’)	Annealing temperature (degree celsius)	Amplicon size (base pair)
hly A	F: AACAAGGATAAGCACTGTTCTGGCT R: ACCATATAAGCGGTCATTCCCGTCA	63	1177
fim H	F: TGCAGAACGGATAAGCCGTGG R: GCAGTCACCTGCCCTCCGGTA	63	508
pap C	F: GTGGCAGTATGAGTAATGACCGTTA R: ATATCCTTTCTGCAGGGATGCAATA	63	200
iut A	F: GGCTGGACATCATGGGAACTGG R: CGTCGGGAACGGGTAGAATCG	63	302
cnf 1	F: AAGATGGAGTTTCCTATGCAGGAG R: CATTCAGAGTCCTGCCCTCATTATT	63	498
sat	F: ACTGGCGGACTCATGCTGT R: AACCCTGTAAGAAGACTGAGC	55	387

The master mix for each PCR reaction volume contained 10 pmol of forward and reverse primers (Sigma-Aldrich), 10 mm deoxyribonucleotide triphosphate (Takara), 5 U Taq polymerase (Takara), and 10 buffer with MgCl2 (Takara). Each PCR reaction volume also contained 2 L of the deoxyribonucleic acid (DNA) template added to the master mix. 

Amplification of the virulence genes was performed using a thermal cycler (Veriti 96 well; Applied Biosystems) under the following conditions: initial denaturation at 95°C for four minutes, followed by 32 cycles of denaturation at 94°C for 30 seconds, annealing based on the primer employed (Table 1) for 30 seconds with an extension at 72°C for 50 seconds, and a final extension for one cycle at 72°C for five minutes. The PCR product was then run on a 1.5% agarose gel stained with ethidium bromide for detection of the amplified virulence gene fragment.

DNA sequencing 

PCR-positive amplicons for each virulence gene detected were purified and sequenced. The sequenced strains for each gene served as positive controls. Sequencing was done by using the Sanger AB13730 XL DNA analyzing instrument (AgriGenome; Kerala). Using the Bioedit sequence programme (product version 7.0.5.3), the nucleotide sequences were aligned, and they were then compared with the basic alignment search tool offered on the National Centre for Biotechnology Information website (www.ncbi.nIm.nih.gov). The nucleotide sequences that were analyzed were submitted to the GenBank and the accession numbers were obtained.

The DNA sequences of these virulence genes were submitted to GenBank, and the following accession numbers were obtained: MZ420493-iut A, MZ501821-sat, MZ198898-fim Hiut MZ460390-cnf 1 and MZ465579- hly A.

The statistical analysis was performed using IBM SPSS Statistics for Windows, Version 16 (Released 2007; IBM Corp., Armonk, New York, United States). To determine the significance of the link between virulence genes and antibiotic resistance, a univariate analysis was conducted. Proportions were compared using the chi-square test and the differences were considered significant if P-value ≤0.05 was considered statistically significant.

## Results

A total of 150 isolates were included in this study; of them, 80 (53%) were from female patients and 70 (46%) were from male patients.

Molecular methods

Gene Profile

PCR analysis revealed that of the 150 *E. coli *included in the study, virulence genes were detected in 112 (74%). Overall, *fim H* gene was detected in 86 (76.78%) followed by i*ut A* gene in 64 (57.14%), *sat* gene in 50 (44.64%), *cnf* gene in 29(25.89%) and h*ly A* gene 2(1.78%). The p*ap C* gene was not detected in any of the isolates depicted in Table 2.

**Table 2:** Prevalence of virulence genes among the isolates

Genes	Prevalence (Total = 150) (%)
fim H	86 (76.78)
Iut A	64 (57.14)
cnf 1	29 (25.89)
hly A	2 (1.78)
sat	50 (44.64)
pap C	0

Only a single virulence gene was detected in 44 (29.33%) isolates. Out of 44 the virulence genes that occurred singly were f*im H *(34), i*ut A* (5), *SAT* (4) and *cnf-1 *(1). The *hyl A* gene did not occur solely but it coexisted with other virulence genes like *fimH, iutA, sat,* and *cnf* genes.

Gel Electrophoresis of PCR for Detecting Virulence Gene in UPEC

**Figure 1:**
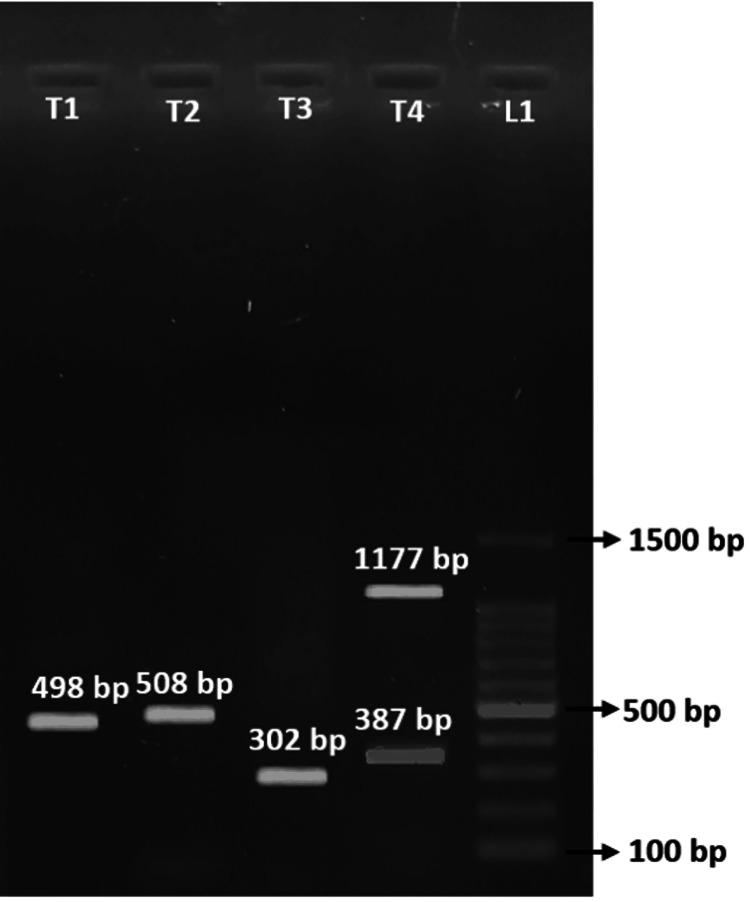
Image of gel electrophoresis of virulence genes detected

The band at 498bp (T1) represents the presence of *cnf* 1 gene, the band at 508bp (T2) represents the presence of *fim H* gene, the band at 302bp (T3) represents the presence of *iut A* gene, the band at 387bp (T4) represents the presence of sat gene, and the band at 1177bp (T4) represents the presence of *hly A* gene. L1 is the 100bp ladder (Figure 1, Table 3).

**Table 3:** Genes and their corresponding base pairs

Genes	Base pair
fim H	508 bp
cnf 1	498 bp
hly A	1177bp
Iut A	302bp
sat	387bp

Of the 112 isolates that carried virulence genes, 68 (60.71%) carried more than one gene in various combinations. Some combinations of virulence genes were found in single isolates only. Thirty-eight (25.33%) of the study isolates did not anchor any of the virulence genes looked for in this study. 

Antimicrobial susceptibility pattern

The Kirby-Bauer disc diffusion method revealed the resistance pattern for the antibiotics as follows: amikacin 19 (12.6%), gentamicin 28 (18.6%), fosfomycin 3 (2%), ampicillin 122 (81.3%), cefotaxime 103 (68.6%), ciprofloxacin 127 (84.6%), cotrimoxazole 93 (62%), nitrofurantoin 11 (7.3%), piperacillin-tazobactam 32 (21.3%), and imipenem 18 (12%).

*iut A *gene occurred more frequently in isolates resistant to ampicillin, ciprofloxacin, cotrimoxazole, and piperacillin-tazobactam. Similarly, the *sat *gene was found in isolates resistant to gentamicin, ampicillin, cefotaxime, and cotrimoxazole. No statistical significance was found between other virulence genes and antimicrobial resistance. This is depicted in Table 4.

**Table 4:** Relationship between virulence genes and antimicrobial resistance The statistical significance between the virulence genes and the antimicrobial resistance is compared with P-values for each antibiotic individually.

Antibiotics		Virulence genes					
		fim H (86)	iut A (64)	hyl A (2)	pap C	cnf-1 (29)	sat (50)
Amikacin	Sensitive	74	59	2	-	27	41
	Resistant	12	5	0	-	2	9
	P-value	0.5836	0.1309	-	-	0.3091	0.1704
Gentamicin	Sensitive	69	53	2	-	24	35
	Resistant	17	11	0	-	5	15
	P-value	0.6886	0.6886	-	-	0.8265	0.0139
Fosfomycin	Sensitive	85	63	2	-	29	48
	Resistant	1	1	0	-	0	2
	P-value	0.4145	0.7429	-	-	0.7159	0.2521
Ampicillin	Sensitive	22	6	0	-	4	4
	Resistant	74	58	2	-	25	46
	P-value	0.0809	0.0153	-	-	0.4560	0.0240
Cefotaxime	Sensitive	23	18	0	-	8	9
	Resistant	63	46	2	-	21	41
	P-value	0.1617	0.4654	-	-	0.6286	0.0173
Ciprofloxacin	Sensitive	12	5	0	-	6	4
	Resistant	74	59	2	-	23	46
	P-value	0.5872	0.0336	-	-	0.3758	0.0872
Cotrimoxazole	Sensitive	30	15	2	-	10	13
	Resistant	56	49	0	-	19	37
	P-value	0.3626	0.0019	-	-	0.6642	0.0343
Nitrofurantoin	Sensitive	81	61	2	-	28	42
	Resistant	5	3	0	-	1	8
	P-value	0.4121	0.2925	-	-	0.3872	0.3987
Piperacillin/Tazobactam	Sensitive	67	56	2	-	24	38
	Resistant	19	8	0	-	5	12
	P-value	0.7924	0.0261	-	-	0.5505	0.5734
Imipenem	Sensitive	75	58	2	-	26	42
	Resistant	11	6	0	-	3	8
	P-value	0.7300	0.3962	-	-	0.7604	0.2904

Of the 150 isolates, 103 were ESBL producers. Among 112 isolates that carried the virulence genes, 79 were ESBL producers. The occurrence of sat genes and ESBL production was statistically significant. There was no difference in the distribution of other genes fim H, cnf-1, and hly A among ESBL and non-ESBL producers (Table 5).

**Table 5:** Distribution of virulence genes among ESBL producers ESBL: Extended spectrum beta-lactamase producer

	Virulence genes (+)	Virulence genes (-)	P-value
ESBL producer	79	24	0.5190
Non-ESBL producer	33	14	

Of the 150 isolates, 18 were carbapenem-resistant. Among the 112 isolates that carried the virulence genes, 14 were carbapenem-resistant. There was no difference in the distribution of any genes among the CRE and non-CRE (Table 6).

**Table 6:** Distribution of virulence genes among carbapenem-resistant isolates CRE: Carbapenem-resistant *Escherichia coli*

	Virulence genes (+)	Virulence genes (-)	P-value
CRE	14	4	0.97234
Non-CRE	98	34	

## Discussion

A comprehensive understanding of the virulence markers of UPEC strains, particularly in hospitalized patients, is essential to monitor the pathogenicity trends of strains responsible for UTIs. In this study, *E. coli* isolated from urine samples of 150 inpatients with pyelonephritis, cystitis, and urosepsis were analyzed for the presence of six virulence genes (*fim H, iut A, hly A, pap C, cnf 1,* and *sat*) and their associated antibiotic susceptibility profiles.

The present study found that 74.66% of the isolates harbored at least one virulence gene, with *fim H (*77%) being the most frequently detected, followed by *iut A* (57%) and *sat* (45%). The high prevalence of *fim H* is significant, as it encodes for type 1 fimbriae, essential for *E. coli* adhesion to host cells in the urinary tract.* fim H *is a critical factor in initiating and establishing UTIs. This aligns with other studies where *fimH *was consistently reported as the most prevalent, in UPEC isolates, regardless of the geographical location or study population. One study in Iran found *fim H* in 99.2% of UPEC isolates [5]. Another study in Nepal showed that 52.3% of UPEC isolates were positive for *fim H*, compared to 8.5% in the control group [2]. A study in Mexico found that *fimH* was present in 86 % of isolates [4]. A meta-analysis of 13 studies also identified *fim H* as one of the most prevalent virulence factors 75.3% [3]. The consistently high prevalence of *fim H* across studies further signifies its importance as a key target for developing strategies to combat UTI.

*iut A* (57%) and *sat* (45%) were the other prevalent genes in the present study. A Mexican study reported iut A in 54.2% and sat in 26.2% of UPEC isolates. The study examining UPEC and APEC isolates in China found iut A in 83% of UPEC and 90% of APEC isolates, while sat was less prevalent, detected in 46.6% of UPEC isolates [4,6]. The low prevalence of *hly A* 2% in the present study contrasts with the study by Shah et al., who found hemolysin production in 32% of UPEC isolates, reflecting variability in virulence expression [2]. The *hly A* gene, encoding the toxin α-hemolysin, also shows a wide range of prevalence across the studies, ranging from 7.4% to 31.25% [4,7]. 

Notably, the *pap C* gene that encodes for pyelonephritis-associated pilus, commonly linked to pyelonephritis, was absent in all isolates, differing from findings reported in other geographical regions. Reported rates of occurrence of *pap C* ranged from 26 to 62%, indicating substantial variability [6,7].

Thus, variation in the virulence gene profile could be attributed to regional differences in UPEC strains, the types of UTIs included in the studies (cystitis, pyelonephritis, urosepsis), or the specific patient populations studied.

The study on antimicrobial resistance revealed varied proportions of resistance among various groups of drugs. Higher resistance rates were observed for ampicillin (81.3%), ciprofloxacin (84.6%), cotrimoxazole (62%), and extended-spectrum cephalosporins (68.6%). One study reported a resistance rate of 74.3% for aminopenicillins among UPEC isolates, highlighting the widespread resistance to this class of antibiotics [3,4].

High resistance to ciprofloxacin (84.6%) and cotrimoxazole (62%) parallels findings by Shah et al., who reported similar resistance levels in UPEC isolates [2]. Many reports have been published on the high rate of resistance to fluoroquinolones, particularly ciprofloxacin, which was an effective treatment option for UTI. One study noted that fluoroquinolone-resistant UPEC represented 31.3% of isolates among hospitalized patients in the US between 2007 and 2010 [8]. A meta-analysis of 13 studies found a pooled resistance rate of 49.4% for quinolones [3]. Resistance to cotrimoxazole, another commonly prescribed antibiotic for UTIs, is also a significant concern. A study in Iran found that 45% of UPEC isolates were resistant to cotrimoxazole [1].

Among the isolates, 68% were ESBL producers. The rise of ESBL-producing UPEC strains is a significant driver of resistance to extended-spectrum cephalosporins. This resistance presents a serious therapeutic challenge, as it limits treatment options and often requires the use of carbapenems, which are reserved for more severe infections. In many studies, the ESBL production among UPEC ranged from 26.9% to 53% [5,7].

The relationship between virulence and antibiotic resistance in UPEC is complex and not fully understood. Some studies suggest that there is a positive correlation between virulence and resistance, while others suggest that there is a negative correlation. A possible explanation for a positive correlation is that virulence factors may help UPEC to survive in the presence of antibiotics. Biofilm formation can protect UPEC from the effects of antibiotics. Additionally, some virulence factors, such as siderophores, can help UPEC acquire iron, which is essential for bacterial growth. This may give UPEC a competitive advantage in the presence of antibiotics, which can limit the availability of iron. In some strains, both the virulence and resistance genes may be located on the same plasmids, resulting in their simultaneous transfer [1,3].

However, some studies have also shown a negative correlation between virulence and antibiotic resistance. One study found that quinolone-resistant UPEC strains were less likely to encode for certain virulence genes, such as *sfa, hly*, and *cnf 1*. This suggests that the acquisition of quinolone resistance may come at the cost of reduced virulence [1-3]. It has been reported that the UPEC strains lose hemolytic capacity in subinhibitory concentrations of ciprofloxacin, showing a partial or total loss of the pathogenicity island (PAI) containing the *hly* and *cnf-1* genes. It has been reported that quinolone-resistant UPEC isolates carried virulence factor genes related to their ability to invade the urinary tract. Virulence factors, like hemolysin, aerobactin,* cnf-1,* and *sat,* are chromosomally encoded in the PAIs, which can be deleted from the chromosome spontaneously. Quinolones can act by increasing the deletion and transposition of DNA regions during the development of quinolone resistance facilitated by exposure to quinolones [3].

In the present study, a significant association between the occurrence of *sat* genes and ESBL production was observed. A study in Iran found that the presence of *papGII, iut A*, and PAI markers was significantly associated with ESBL production. Conversely, a study in India found that the presence of multiple virulence genes was significantly higher in non-ESBL strains than in ESBL-producing strain which examined for the *hly, pap C*, and *cnf-1* genes [5,9]. 

In this study, 18 (12%) of the isolates were CRE. A recent study from Egypt reported a rate of 17 (13.07%), in Turkey and Iran it was 11 (22%) and 7%, respectively [10-12]. In the present study, there was no significant difference observed in the distribution of virulence genes between CRE and non-CRE isolates, indicating that resistance and virulence might have evolved independently. A study by Shah et al. observed no statistically significant association between major virulence genes (*fim H, pap C, hly A*) and carbapenem resistance in UPEC isolates. This aligns with the present findings, highlighting that while resistance mechanisms such as the production of carbapenemases confer survival advantages against antibiotics, they do not inherently enhance or diminish virulence factor expression. 

The findings of this study suggest that virulence factors and resistance mechanisms may not always intersect but can independently influence clinical outcomes. 

Limitations

Only few common virulence genes were studied. The limited sample size and the limited number of genes could have hindered the statics in their association with various classes of antibiotics. The antibiotic resistance coding genes could also be looked for which might have brought accurate associations and might have supported the study.

## Conclusions

The study reveals that f*im H *is the most prevalent virulence factor among UPEC and occurrence of multiple virulence genes within individual isolates. The study reports a very high prevalence of ESBL producers as well as a concerning carbapenem resistance among isolates. Despite the high resistance rates, the study found no significant association between virulence gene presence and antimicrobial resistance profile. Further investigation is essential to explore the potential relationships between resistance and specific virulence mechanisms among UPEC. This knowledge will be vital for developing effective strategies to combat the growing threat of multidrug-resistant UPEC infections.
